# Incidental conformational switching in an allosteric enzyme

**DOI:** 10.64898/2026.08.04.741757

**Published:** 2026-08-07

**Authors:** Paul J. Sapienza, Darex J. Vera-Rodriguez, Trevor R. Mileur, Andrew L. Lee

## Abstract

The classical understanding of allostery was initially grounded in two-state models, such as MWC and KNF, where structure and function are inextricably linked through transitions between low-(T) and high-affinity (R) states. Here, we show Yeast chorismate mutase (CM) provides a vivid example of the growing list of exceptions to the traditional T vs R two-state allosteric paradigm. While CM exhibits dynamic sampling of the R-state in the presence of the activator tryptophan (Trp), suggesting a conformational selection (CS) mechanism, we present multiple instances where conformational status and catalytic activity are decoupled. Using NMR spectroscopy and kinetic assays, we identify CM variants that reside almost exclusively in the T conformation can exhibit maximal activity, while others that predominantly occupy the R conformation are weakly active. Quantitative comparison of experimental data with a parameterized CS model reveals deviations of up to two orders of magnitude, ruling out the simplest two-state model for substrate affinity modulation in this system. We propose that the observed T-to-R switching in CM is “incidental”, a byproduct of an evolved energy landscape that allows access to the substrate-bound pose but does not mechanistically determine affinity. Our findings suggest that allosteric regulation in CM may instead be driven by local features of the ground-state ensemble, which operate independently of global T/R status. This work further highlights an emerging view that the mere observation of a pre-sampled active conformation does not sufficiently prove a two-state mechanism and further underscores the need for deeper ensemble-based perspectives in protein engineering and allostery.

## Full Text Availability

The license terms selected by the author(s) for this preprint version do not permit archiving in PMC. The full text is available from the preprint server.

